# Detecting representative characteristics of different genders using intraoral photographs: a deep learning model with interpretation of gradient-weighted class activation mapping

**DOI:** 10.1186/s12903-023-03033-8

**Published:** 2023-05-25

**Authors:** Yimei Zhou, Fulin Jiang, Fangyuan Cheng, Juan Li

**Affiliations:** 1 State Key Laboratory of Oral Diseases, National Center of Stomatology, West China Hospital of Stomatology, National Clinical Research Center for Oral Diseases, Sichuan University, 14#, 3rd Section, Renmin South Road, Chengdu, 610041 China; 2 Chongqing University Three Gorges Hospital, Chongqing, 404031 China; 3 Chengdu Boltzmann Intelligence Technology Co., Ltd, Chengdu, China

**Keywords:** Deep learning, Sex characteristics, Individual oral treatment

## Abstract

**Background:**

Sexual dimorphism is obvious not only in the overall architecture of human body, but also in intraoral details. Many studies have found a correlation between gender and morphometric features of teeth, such as mesio-distal diameter, buccal-lingual diameter and height. However, it’s still difficult to detect gender through the observation of intraoral photographs, with accuracy around 50%. The purpose of this study was to explore the possibility of automatically telling gender from intraoral photographs by deep neural network, and to provide a novel angle for individual oral treatment.

**Methods:**

A deep learning model based on R-net was proposed, using the largest dataset (10,000 intraoral images) to support the automatic detection of gender. In order to reverse analyze the classification basis of neural network, Gradient-weighted Class Activation Mapping (Grad-CAM) was used in the second step, exploring anatomical factors associated with gender recognizability. The simulated modification of images based on features suggested was then conducted to verify the importance of characteristics between two genders. Precision (specificity), recall (sensitivity) and receiver operating characteristic (ROC) curves were used to evaluate the performance of our network. Chi-square test was used to evaluate intergroup difference. A value of p < 0.05 was considered statistically significant.

**Results:**

The deep learning model showed a strong ability to learn features from intraoral images compared with human experts, with an accuracy of 86.5% and 82.5% in uncropped image data group and cropped image data group respectively. Compared with hard tissue exposed in the mouth, gender difference in areas covered by soft tissue was easier to identify, and more significant in mandibular region than in maxillary region. For photographs with simulated removal of lips and basal bone along with overlapping gingiva, mandibular anterior teeth had similar importance for sex determination as maxillary anterior teeth.

**Conclusions:**

Deep learning method could detect gender from intraoral photographs with high efficiency and accuracy. With assistance of Grad-CAM, the classification basis of neural network was deciphered, which provided a more precise entry point for individualization of prosthodontic, periodontal and orthodontic treatments.

**Supplementary Information:**

The online version contains supplementary material available at 10.1186/s12903-023-03033-8.

## Background

In treatment of the oral and perioral area, the correct arrangement of hard and soft tissue is important for maintenance of good function and aesthetic appearance, which is in strong association with gender. Sexual dimorphism is obvious not only in the overall architecture of human body, but also in intraoral and perioral details. During the last two decades, many studies have attempted to understand the correlation existing between gender and details both intraoral and perioral. For example, prosthodontists believed that the characteristics of men and women should be reflected respectively through the shape and color of dentures while designing [1]. For periodontists, studying of sexual dimorphism in morphology and thickness of gingiva lent itself to planning of periodontal surgery and prediction of clinical outcome [2, 3]. Apart from practice in clinical dentistry, various oral and perioral features such as morphology, dental arch length and width, and soft tissue like palatal and lips, were also being used to determine sex of a particular population in accidents and mass disasters in forensic odontology [4, 5].

For both clinical dentistry and in forensic odontology, it’s of great importance to get comprehensive information on oral sexual dimorphism. However, previous studies usually focused on more noticeable features partially. In fact, as an ensemble of hard and soft tissues, more details are hidden in the oral cavity, and intraoral photograph is a valuable information carrier. As to forensic odontology, intraoral photograph is the most accessible form of record during identification. For clinical dentistry, it is an essential part of an ideal treatment goal. Results of previous studies showed that humans were limited in their ability to extract gender information from intraoral photos. Accuracy around 50% can only be reached when intraoral photographs were the only clue to discriminate sex both for laymen and dental specialists [6–8]. There is no denying that morphometric difference exists between two genders, but results of previous studies indicated that it couldn’t be recognized by human eye in photographs of front teeth region, probably because of the tiny scale or unnoticed gender difference.

In the last two decades, computer-aided detection has developed significantly in medicine. Because of remarkable advantage in image processing, convolutional neural networks (CNNs) and variations of pre-trained CNNs have been proven to be successful in the dental field [9–12]. However, another deep neural network in mainstream, recurrent neural network (RNN), which exhibits serial dynamic behavior in the task, hasn’t received enough attention before. Since a strong correlation exists between adjacent slices in cone-beam computed tomography (CBCT), extracting intra-slice and inter-slice contexts is the main application of RNN in the dental filed [13]. R-net is an extraction-based reading comprehension model developed in 2017, based on RNN [14]. It solves tasks in a way similar to us doing reading comprehension: reading the input several times, linking questions with corresponding part of the input, aggregating information collected from the input, and selecting the final answer after predicting the probability distribution the start and end position of the answer in iteration. By applying R-net to dental image processing, many visual tasks can be simplified as natural language problems in a novel way. Although deep learning methods may show great promise in detecting differences in dental images, the lack of ability to interpret deep neural network models makes it difficult to get extra information from the results. Grad-CAM is a targeted technique creating heatmaps showing the importance of different regions in the input image to the deep neural network with different colors. It provides visual explanations of what the neural network sees and how it understands when making a decision.

Here, a pioneering study is presented using R-net to explore the possibility of identifying gender with front view intraoral photograph based on a dataset from 10,000 patients. Comprehensive evaluation showed that the R-net model based on a typical deep learning structure was able to classify male and female with clinical acceptable accuracy, which provides possibility to improve the efficiency of identification in mass disasters. With assistance of Grad-CAM, the classification basis of neural network was reversely analyzed, which provided a more precise entry point for individualization of prosthodontic, periodontal and orthodontic treatments.

## Methods

### Data collection and processing

This study was conducted in the Department of Orthodontics in West China Hospital of Stomatology Sichuan University, and was approved by the local institutional review board/ethics committee (WCHSIRB-D-2019-120). Written consent and approval of the patients concerned or patients’ legal guardians were obtained before their participation in the study. The dataset included intraoral front view photographs taken from 10,000 patients, which are randomly chosen from the image archive of the hospital from January 2020 to December 2020. These photos were all captured at the same angle, same settings of the camera and flash lamp, using the same device by the same photographer. Images were excluded from the study if the patient (a) had received or was under orthodontic treatment, (b) had prosthetic reconstructions such as fillings, veneers, crowns or bridges, (c) had fluorosis or other diseases or experience which may affect the natural appearance of teeth, (d) had fewer or more teeth than usual (the third molars were not included). Aside from raw image data, These photographs were also cropped to analyze importance of teeth. For anonymity, patients concerned were coded and only gender and age information were extracted.

### Neural network training

The gender detection model was built on R-net framework and trained using raw image data or cropped image data to further fine-tune the current framework based on transfer learning techniques. The details of this procedure can be summarized into two parts. First, the basic R-net framework was trained with the visual object classes dataset to intelligently propose regions of interest (ROI) which may contain data points pertinent to the task at hand (classification according to gender in this study). Second, identifications made by the artificial intelligence (AI) model were compared with the recorded gender of each patient to allow the AI model learn from its mistakes. The trained R-net was then validated against an independent dataset, and performance metrics were derived.

### Performance analysis

The dataset of 200 images was used to evaluate the performance of R-net model, which was not included in training set. Precision (specificity), recall (sensitivity) and ROC curves are the most common metrics for binary classification problem. As is true with any other evaluation algorithm, these metrics are evaluated in comparison to the ground truth, namely recorded gender in this study. For the detection task, the ground truth and system annotations were deemed to agree if they intersected substantially. The remaining errors were composed of two types (take photographs with annotations of male by AI model as an example): false positive results, where female patients were annotated, and false negative results, where male patients were missed. The categorical data were analyzed using chi-square test to evaluate performance of R-net model. A value of p < 0.05 was considered statistically significant. SPSS software (version 24, Chicago, IL, USA) was used for the statistical analysis.

## Results

**Fig. 1:**
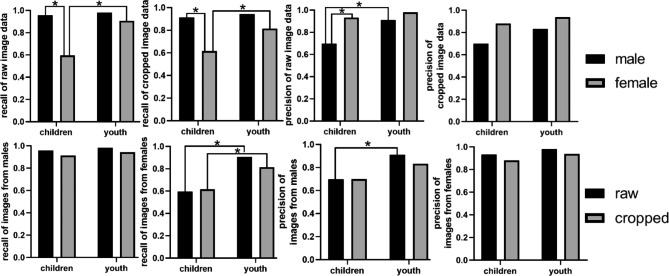
Performance of the R-net model in testing set when dealing with uncropped (raw) images and cropped images where perioral soft tissues were removed. Results showed that the R-net model exhibited higher recall (sensitivity) in male detection and higher precision (specificity) in female detection in contrast to performance in detection of the other gender. For cropped images and images from individuals under 15 years old, performance of the R-net model was decreased. * p < 0.05

Demographic and clinical characteristics of the patients included in the study are shown in Additional Table 1. A total of 10,000 front view intraoral photographs from 4786 males and 5214 females aged between 5 and 56 years (median age 19 years) were analysed as training set. The testing set included 200 intraoral photographs from 99 males and 101 females aged between 7 and 44 years (median age 16 years) which were not included in the training set. The R-net model yielded a relatively high performance in the detection of gender (Additional Fig. 1). An overall accuracy of 86.5% and 82.5% was achieved in raw image data group and cropped image data group respectively. Results showed that the R-net model exhibited higher sensitivity in male detection and higher specificity in female detection in contrast to performance in detection of the other gender. To further evaluate the accuracy of R-net model in patients of different ages, the testing set was divided into 7–14 years old as children group and 15–44 years old as youth group, considering the growth and development pattern of children. Results showed that performance of R-net model was better in 15–44 years old group than in 7–14 years old group. In 7–14 years old group, an overall accuracy of 77.4% and 76.3% was achieved in raw image data group and cropped image data group respectively. The detailed data are presented in Tables 1 and 2; Fig. 1.

**Table 1:** Performance of the R-net model for male detection in the testing set

Group	Actual Male		Actual Female		Sensitivity	Specificity
	True Positive	False Negative	True Negative	False Positive		
Uncropped Children Group	44	2	28	19	0.957	0.698
Cropped Children Group	42	4	29	18	0.913	0.7
Uncropped Youth Group	52	1	49	5	0.981	0.912
Cropped Youth Group	50	3	44	10	0.943	0.833
Uncropped Testing Samples	96	3	77	24	0.970	0.762
Cropped Testing Samples	92	7	73	28	0.929	0.723

**Table 2:** Performance of the R-net model for female detection in the testing set

Group	Actual Female		Actual Male		Sensitivity	Specificity
	True Positive	False Negative	True Negative	False Positive		
Uncropped Children Group	28	19	44	2	0.596	0.933
Cropped Children Group	29	18	42	4	0.617	0.879
Uncropped Youth Group	49	5	52	1	0.907	0.98
Cropped Youth Group	44	10	50	3	0.815	0.936
Uncropped Testing Samples	77	24	96	3	0.762	0.970
Cropped Testing Samples	73	28	92	7	0.723	0.929

**Fig. 2:**
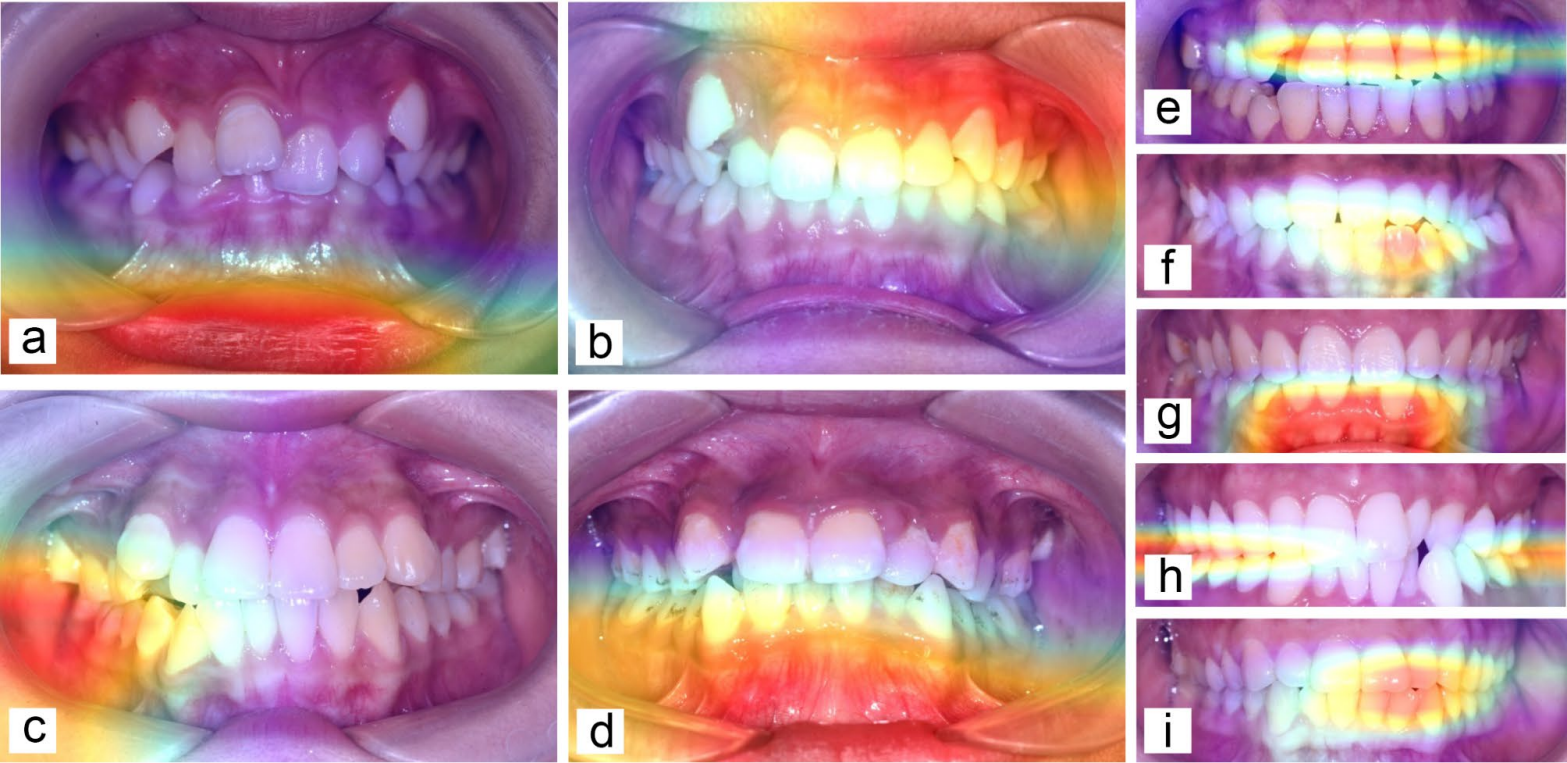
Representative Gaussian heatmaps demonstrating interested regions of the R-net model which were marked with red color when dealing with uncropped and cropped images. Figure a–d showed that the R-net model paid more attention to (a) lip, (b)areas covered by soft tissue around maxillary teeth, (c) areas covered by soft tissue around lower posterior teeth, and (d) areas covered by soft tissue around lower anterior teeth when dealing with uncropped images. Figure e–i showed that the R-net model paid more attention to (e) upper anterior teeth, (f) lower anterior teeth, (g) marginal gingiva of lower anterior teeth, (h) occlusal region of posterior teeth, and (i) occlusal region of anterior teeth

To reversely analyze the classification basis of R-net method, Grad-CAM was conducted. In Fig. 2, some heatmaps of the R-net detection method were randomly chosen as representative results, where attraction of the R-net was marked by different colors. It can be seen that the high-weights of red color are regions detected by the model to be the most important basis which can suggest gender of the individual. Through analyzing the heatmap images of the testing set, it’s found out that for uncropped image data, the R-net model focused more on lips, areas covered by soft tissue around maxillary teeth, around lower posterior teeth and around lower anterior teeth, as shown in Fig. 2a–d. When perioral tissues were removed in cropped images, the R-net model tended to pay more attention to center of the images, such as upper anterior teeth, lower anterior teeth, occlusal region of anterior teeth, and occlusal region of posterior teeth, as shown in Fig. 2e–i. The results showed that deep learning method can extract features in photographs which were highly related to gender independently, and build comprehensive correlation between multiple features and gender of an individual.

## Discussion

In order to understand oral sexual dimorphism from a more comprehensive way, this study focused on detecting gender from intraoral photographs. It is an interesting topic beyond the capabilities of human experts, but neural networks have not yet been applied to this area. This study demonstrated the potential of R-net in gender determination using intraoral front view photograph. It can be used as a gender determination tool to improve the efficiency of identification at the scene of mass disasters. In such situation, features on body surface are often difficult to identify, but intraoral traits are relatively well preserved. With the assistance of R-net model, basic identity determination can be made without any additional measurements. It may also improve individualized treatment goals by helping recognizing sexual dimorphism in intraoral traits and assess the outcome of some dental treatments, because the ideal oral treatment results should be in line with the original physiological characteristics of the patient.

It’s found out that in uncropped images, the R-net model focused not on teeth but on areas covered by soft tissue. When attention of the neural network was adjusted to the teeth by cropping, the accuracy of the R-net model for gender determination decreased. To our knowledge, in front view intraoral heatmaps, appearance of the high-weight red regions covered by soft tissues are mainly affected by the gingiva, alveolar bone, and teeth roots. As for gingiva, whether gender has significant influence on the morphology and color of it is pending further discussion [15–18]. In contrast to gingiva, morphology of alveolar bone has been proved related to gender. A meta-analysis by Shafizadeh et al. [19] indicated that when gender was the only factor that taken into account, thickness dimorphism was detected in most of the variables of the alveolar dimensions. When individuals were further divided according to jaw relationships, differences in anterior alveolar thickness were more significant than those in posterior alveolar thickness [20, 21]. Apart from gingiva and alveolar bone, buccal-lingual inclination of teeth also influences morphology of intraoral soft tissues. It has been pointed out that the inclinations of upper and lower incisors differ in different gender with the same vertical craniofacial relationship [22, 23]. As for posterior teeth, it has been reported that the buccal-lingual inclination of them is quite different between males and females; moreover, this relationship changes with the increasing age [24, 25]. Wilson GH reported the lateral inclination of the posterior teeth in normal untreated patients, namely buccal inclination of the upper posterior teeth and lingual inclination of lower posterior teeth [26]. The existence of appropriate occlusal curvature allows for proper masticatory function and avoids the occlusal interference caused by accentuated curve. However, in orthodontic treatment, clinicians tend to employ the same approach to treat different patients because of simplicity, ignoring the individuality of different people, such as the gender dimorphism found in this study. Based on the study from Weinstein et al., the position of each tooth was the result of equilibrium with surroundings including the bucco-labial muscle, the tongue and the bone [27]. Accordingly, findings of this study indicated that the choice and expression of torque may need to be tailored to individual patients.

During analysis of uncropped heatmaps, a clear trend was observed that the gender dimorphism of mandibular areas covered by soft tissue was more significant than that of the upper areas. In heatmaps of different genders, both anterior and posterior mandibular areas covered by soft tissue can be employed separately as classification basis, but in maxillary region, anterior and posterior areas covered by soft tissue must be employed as a whole. One possible reason is that the gender dimorphism in thickness of lower anterior alveolar bone is greater than that of upper anterior alveolar bone [19]. Also, the thickness of upper anterior gingiva is greater than that of lower anterior gingiva [2], which may blanket the difference in morphology of alveolar bone caused by the inclined incisors in different genders. Though some studies found difference in the contour of maxillary anterior gingiva between two genders [3, 28], the current results suggested that this difference in contour of maxillary anterior gingiva may not be stable enough to tell gender alone, or dental crowding may have influence on this method, which requires further investigation.

In uncropped images, greater weight of lips could be ascribed to wrinkles on the labial mucosa called lip prints, which has been proved different between different genders in contemporary forensics [29, 30]. In images where lips were the only high-weights region, most mistakes were made because females were marked as male by the R-net model, illustrating that rather dominant patterns may exist in males, which was different from present studies [30, 31]. When considering sexual difference in perioral region, another feature which may immediately come to mind is facial hair, which indeed occurred in some images in the training set. However, most of the individuals whose perioral soft tissue was paid attention to by the R-net model were 11–13 years old, which didn’t fit the growth pattern of facial hair. Accordingly, importance of lip prints patterns in gender determination is emphasized. Although in images of this study lips were all stretched by cheek retractors to some extent, usually flat lip prints can be obtained from glasses, cups and cigarettes at the crime scene [32], which prompts that the application of AI methods in gender identification using lip prints is promising in forensic odontology.

In cropped images where lips and basal bone along with overlapping gingiva were eliminated, R-net concentrated on teeth and marginal gingiva. The accuracy reached over 90% when upper and lower anterior teeth were the only interested area. When upper or lower anterior teeth were treated as basis of gender detection separately, the accuracy was similar. When upper posterior teeth, lower posterior teeth or occlusal region of posterior teeth was added as secondary basis, the accuracy got further improvement. This intriguing finding suggests that similar sexual dimorphism in the upper anterior teeth [33, 34] may also exist in the lower anterior teeth, yet often ignored due to weaker visual impression. In fact, for individuals with open bite to moderate overbite, lower anterior teeth can still be exposed from frontal view. In the static smile of individuals with average or low smile lines and most people’s oral movements (such as speaking), the lower anterior teeth also have an impact on aesthetics. Thus, they deserve more attention not only in clinical dentistry but also in forensic odontology. As described before, sexual difference in buccal-lingual inclination of posterior teeth did exist. However, it’s rather strange in heatmaps that most of the red color area only covered the posterior occlusal region, instead of covering the whole inclined crown of teeth. Accordingly, it’s speculated that the underlying basis attributing to gender determination in posterior teeth could be width of dental arch [35] or the occlusal pattern, because similar phenomenon was observed in images involving upper and lower anterior teeth. However no relevant report in the literature has been found. For cropped photographs, image sizes were also reduced, which means the R-net would pay attention to more details with the same training parameters. Although an acceptable accuracy was reached when upper and lower anterior teeth were treated as basis of gender determination in cropped images, the sexual dimorphism of perioral soft tissue is no doubt more visible in front view photographs.

**Fig. 3:**
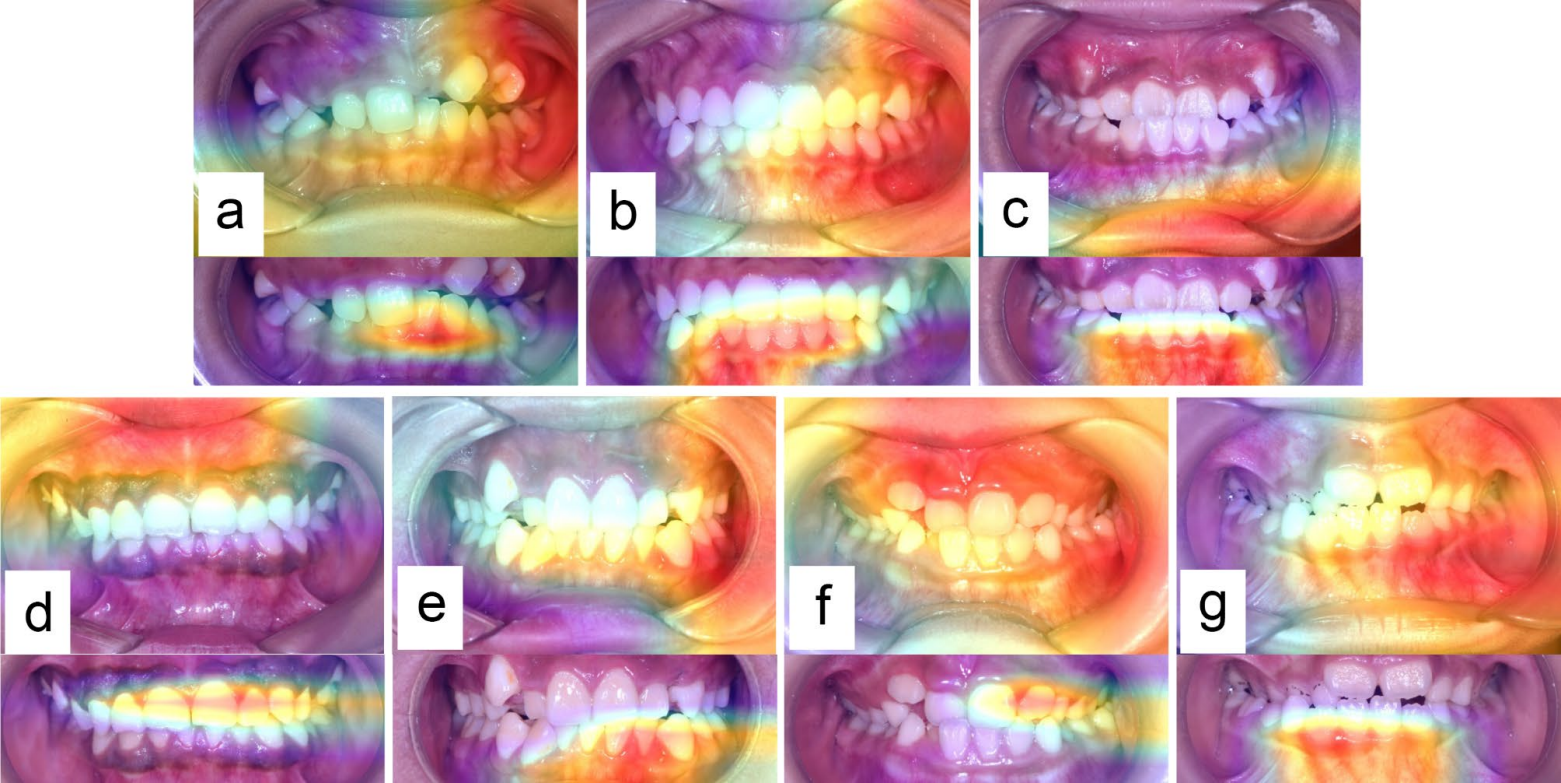
Representative Gaussian heatmaps in 7–14 yrs female group demonstrating area of interest, which were falsely predicted by our network. Figure a–c showed images where mistakes were corrected by cropping. Figure d–e showed images where mistakes occurred after being cropped. Figure f–g showed images which were incorrectly labelled both before and after being cropped

By dividing images into children group (7–14 years old) and youth group (15–44 years old), it’s found out that inaccuracy of R-net model came mainly from photographs of some 7–14 years old females which were detected as males’ incorrectly. Among these images, some mistakes were corrected by cropping. In heatmap of uncropped images, area of interest was focused on canine which was malposed (Fig. 3a) or hadn’t erupted completely (Fig. 3b), or on lips (Fig. 3c). After being cropped, area of interest turned to mandibular incisors, thus detection result was corrected. Some mistakes happened when images were cropped, in which case area of interest was focused on lips or buccal mucosa before, yet led to teeth afterwards. However, because of incompletely erupted teeth (Fig. 3d) or severely crowded mandibular anterior teeth (Fig. 3e), corrected detection was unable to be made. The other images couldn’t be detected correctly both before and after being cropped. Usually, dentition in these photographs had mostly incompletely erupted teeth (Fig. 3f) or anomalous dentition arch (Fig. 3g). To our knowledge, frontal photograph of dentition can provide tremendous amount of information to tell the gender of one subject. Of these features, some are very specific; hence have greater weight in gender detection. However, these features were not stable or obvious at young age, causing the low accuracy in children group.

However, this study has some limitations. [1] Many intraoral features are related to not only the gender of individual, but also vertical and sagittal jaw relationship, such as buccal-lingual inclination of teeth and thickness of lower anterior alveolar bone [19, 20]. However, combination of data from different jaw patterns may conceal the genuine sexual dimorphism because of the unknown prevalence of different jaw patterns in males and females. Detailed research should continue to improve the AI discrimination. [2] Grad-CAM can only narrow down classification basis of neural network to smaller region. More direct methods of interpreting learning need to be developed.

## Conclusion

In conclusion, this study presented and validated a new AI-driven tool for fast and accurate gender detection using front view intraoral photograph, whose performance was better than recorded human experts. It can improve the efficiency of identification at the scene of mass disasters, and it can also be applied to assess the outcome of some dental treatments. With assistance of Grad-CAM, the classification basis of neural network was deciphered, which provided a more precise entry point for individualization of prosthodontic, periodontal and orthodontic treatments. The use of more robust and interpretable systems is an important step for a more precise and personalized dental practice.

## Electronic supplementary material

Below is the link to the electronic supplementary material.

Supplementary Material 1

Supplementary Material 2

## Data Availability

The data that support the findings of this study are available from West China Hospital of Stomatology Sichuan University but restrictions apply to the availability of these data, which were used under license for the current study, and so are not publicly available. Data are however available from the authors upon reasonable request and with permission of West China Hospital of Stomatology Sichuan University.

## Abbreviations

CNN: convolutional neural network
RNN: recurrent neural network
CBCT: cone-beam computed tomography
ROI: regions of interest
AI: artificial intelligence
ROC: receiver operating characteristic
Grad-CAM: Gradient-weighted Class Activation Mapping
