# Supplementary material for: Detecting representative characteristics of different genders using intraoral photographs: a deep learning model with interpretation of gradient-weighted class activation mapping
Source: BMC Oral Health. 2023 May 25;23:327. doi: 10.1186/s12903-023-03033-8 (PMC10214706; doi:10.1186/s12903-023-03033-8)

**Additional Figure 1.** The ROC curves of our model. Class 1 represents male and class 0 represents female.


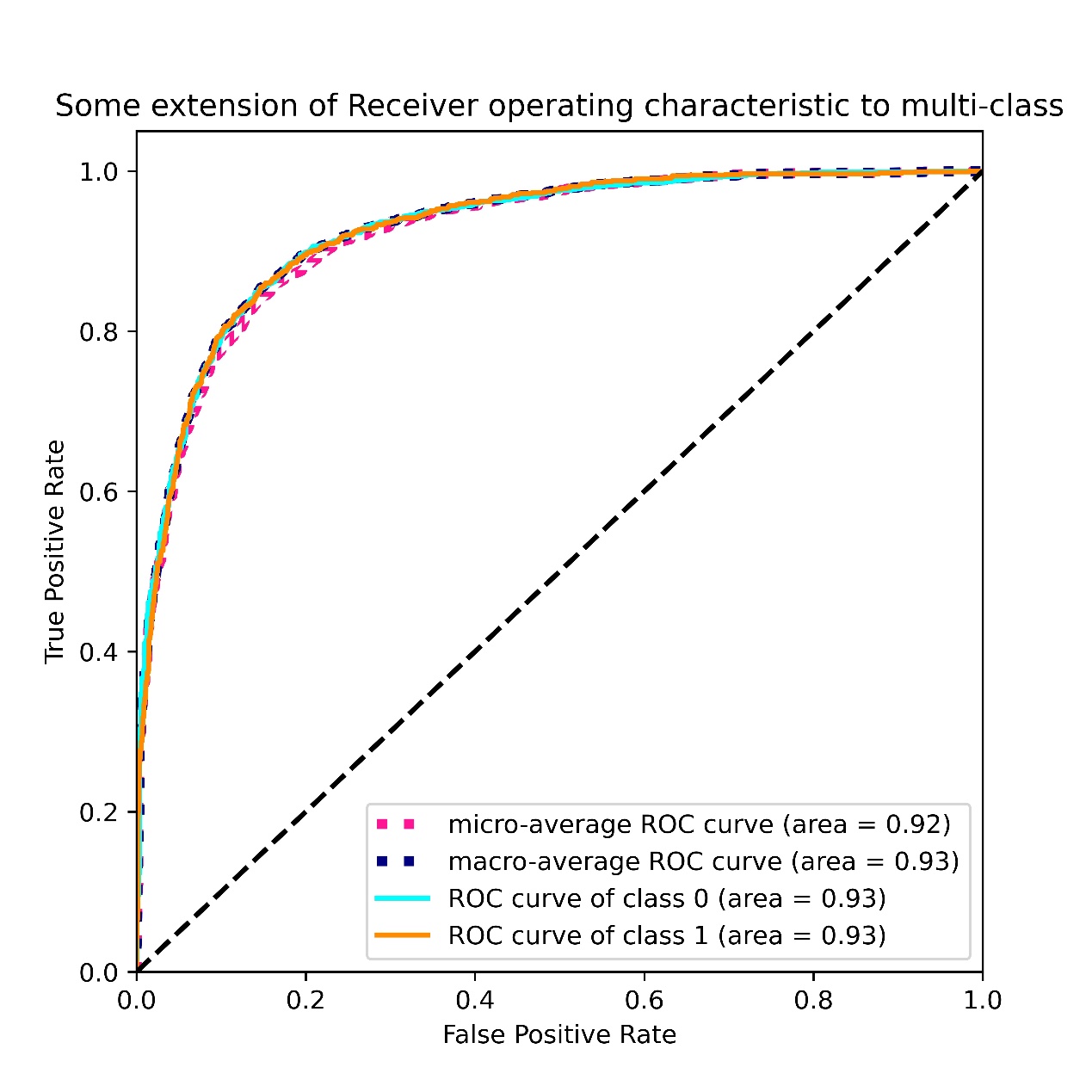

Supplement: Supplementary file 1 — Supplementary Material 1 [file 12903_2023_3033_MOESM1_ESM.docx]
