# Supplementary material for: Detecting representative characteristics of different genders using intraoral photographs: a deep learning model with interpretation of gradient-weighted class activation mapping
Source: BMC Oral Health. 2023 May 25;23:327. doi: 10.1186/s12903-023-03033-8 (PMC10214706; doi:10.1186/s12903-023-03033-8)
Supplement: Supplementary file 2 — Supplementary Material 2 [file 12903_2023_3033_MOESM2_ESM.docx]

**Additional Table 1.** Clinical and demographic characteristics of individuals included.

| Characteristic | Training Set  (n = 10000) | Testing Set  (n = 200) |
| --- | --- | --- |
| Age, median (range), y  Gender, n (%)  Male  Female | 19 (5–56) | 16 (7–44) |
|  |  |  |
|  | 4786 (47.86)  5214 (52.14) | 99 (49.5)  101 (50.5) |
